# Luteolin-7-O-rutinoside alleviates rheumatoid arthritis through downregulation of the PI3K/AKT/NF-κB and cell cycle signaling pathways

**DOI:** 10.3389/fphar.2026.1879862

**Published:** 2026-08-28

**Authors:** Weixi Liu, Xian Jiang, Shanqi Xu, Weiding Cui, Xiaodong Chen, Liqun Wang, Ruiping Liu

**Affiliations:** 1 Department of Orthopedic Surgery, Xinhua Hospital, Shanghai Jiao Tong University School of Medicine, Shanghai, China; 2 Department of Biochemical Engineering, College of Pharmaceutical Engineering and Life Science, Changzhou University, Changzhou, China; 3 Department of Orthopaedics, The Affiliated Changzhou No.2 People’s Hospital of Nanjing Medical University, Changzhou, China; 4 Department of Orthopaedics, The First Affiliated Hospital of Nanjing Medical University, Nanjing, China; 5 Changzhou Medical Center, Nanjing Medical University, Changzhou, China

**Keywords:** cell cycle, fibroblast-like synovial cells, luteolin-7-O-rutinoside, PI3K/Akt/NF-kB, Rheumatoid arthritis

## Abstract

**Background:**

Rheumatoid arthritis (RA) is a chronic autoimmune disease with limited therapeutic options, and current therapies are often associated with considerable adverse effects. Traditional Chinese medicinal plants such as *Cirsium japonicum* contain luteolin-7-O-rutinoside (lut-7-O-rutin), a flavonoid with reported anti-inflammatory activity.

**Aim of the study:**

Lut-7-O-rutin is a flavonoid compound found in various medicinal plants. This study aimed to investigate the therapeutic effects of lut-7-O-rutin on RA and elucidate the underlying mechanisms using both *in vitro* and *in vivo* models, with a particular focus on the phosphatidylinositol 3-kinase/protein kinase B/nuclear factor-κB (PI3K/AKT/NF-κB) and cell cycle signaling pathways.

**Materials and methods:**

*In vitro* experiments, including ELISA, Western blotting, and immunofluorescence assays, were performed to evaluate the anti-inflammatory effects of lut-7-O-rutin on the IL-1β-induced inflammatory response in fibroblast-like synoviocytes (FLSs) derived from rat knee synovium. The effects of lut-7-O-rutin on the PI3K/AKT/NF-κB and cell cycle signaling pathways were also examined. *In vivo* experiments were conducted to assess the therapeutic effects of lut-7-O-rutin administered by gastric gavage in collagen-induced arthritis (CIA) rats.

**Results:**

Lut-7-O-rutin at 3.75 μg/mL significantly reduced the levels of TNF-α, IL-6, and COX-2 in IL-1β-induced FLSs. It also decreased the phosphorylation levels of key components of the PI3K/AKT/NF-κB signaling pathway, thereby attenuating inflammation. In addition, lut-7-O-rutin downregulated the expression of the cell cycle-associated proteins CDK4 and CCND and inhibited FLS proliferation. *In vivo*, 21 days of lut-7-O-rutin administration by gastric gavage resulted in a significant reduction in arthritis scores, organ indices, and serum inflammatory mediator levels in CIA rats. Histopathological analysis showed that lut-7-O-rutin alleviated synovial inflammation, synovial hyperplasia, and bone erosion. Immunohistochemistry (IHC) analysis further showed that lut-7-O-rutin significantly inhibited the expression of key proteins in the PI3K/AKT and cell cycle signaling pathways.

**Conclusion:**

These findings demonstrate that lut-7-O-rutin alleviates joint inflammation and synovial hyperplasia in RA may be mediated through inhibiting the PI3K/AKT/NF-κB signaling pathway and downregulating cell cycle-associated proteins, highlighting its potential as a therapeutic agent for RA.

## 1 Introduction

Rheumatoid arthritis (RA), an autoimmune disease characterized by chronic synovitis, and the progressive destruction of articular cartilage and bone (Yu et al., 2025; Liu and Wei, 2025; Lu et al., 2025), has a substantial impact on patients’ quality of life. The global prevalence of RA is approximately 1% (Luo et al., 2025; Liu et al., 2025). Drug treatment options for RA currently mainly include nonsteroidal anti-inflammatory drugs, disease-modifying anti-rheumatic drugs, such as methotrexate, and biologic agents, such as tumor necrosis factor-α (TNF-α) inhibitors (Nikolova-Ganeva et al., 2025; Tiwari, 2017). More recently, research on RA has explored the use of natural herbal medicines that are effective and less toxic (Wang et al., 2025a; Qiao et al., 2025). Luteolin-7-O-rutinoside (lut-7-O-rutin) is a flavonoid compound found in various medicinal plants, such as honeysuckle and chrysanthemum (Vinayagam and Sudha, 2015). Lut-7-O-rutin has attracted considerable attention due to its core molecule luteolin, which has been shown to have strong anti-inflammatory, antioxidant, and immune-regulating activities (Moualek et al., 2025; Semmarath et al., 2025; Rocchetti et al., 2023). Lut-7-O-rutin exhibits greater water solubility and potentially improved bioavailability compared with luteolin due to its glycoside structure. However, the specific role and molecular mechanisms of lut-7-O-rutin in RA remain unclear.

Here, we performed toxicity assays using lut-7-O-rutin with a purity of 99.1% on rat fibroblast-like synoviocytes (FLSs) to determine an appropriate intervention concentration for subsequent experiments. We confirmed that lut-7-O-rutin exerted significant anti-inflammatory effects in IL-1β-induced FLSs. Transcriptome sequencing of the inflammation model and lut-7-O-rutin treatment groups revealed that the phosphatidylinositol 3-kinase/protein kinase B/nuclear factor-κB (PI3K/AKT/NF-κB) and cell cycle pathways may play important roles in the therapeutic effects of lut-7-O-rutin.

Recent studies examining the pathogenesis and development of treatment strategies for RA have focused on the PI3K/AKT/NF-κB signaling pathway (Liu et al., 2025; Li et al., 2022; Zhuang et al., 2020; Xin et al., 2023; Deng et al., 2026; Liu et al., 2021). Numerous studies have shown that this pathway is abnormally activated in synovial cells from patients with RA, driving the pathological progression of RA by promoting the secretion of inflammatory factors, such as TNF-α, IL-1β, and IL-6, promoting synovial proliferation, and inhibiting cell apoptosis (Zhang et al., 2025; Wang and Liu, 2025b; Zhang et al., 2024). Lut-7-O-rutin is a plant-derived flavonoid compound with multi-target activity and low toxicity, which has shown great potential in regulating inflammatory signaling pathways and is currently a promising focus in drug development (Li et al., 2025; Yuan et al., 2025). Thus, we hypothesized that lut-7-O-rutin may exert therapeutic effects against RA by targeting and inhibiting this pathway.

The current study aimed to investigate the following three aspects. First, we sought to verify the anti-inflammatory effects of lut-7-O-rutin on IL-1β-induced rat FLSs by examining the inhibitory effects of lut-7-O-rutin on inflammatory mediators such as IL-6, TNF-α, and cyclooxygenase-2 (COX-2). Second, we investigated the regulatory effects of lut-7-O-rutin on the phosphorylation levels of key proteins in the PI3K/AKT/NF-κB and cell cycle signaling pathways in IL-1β-induced rat FLSs. Finally, we carried out *in vivo* experiments using a collagen-induced arthritis (CIA) model in rats to evaluate the effects of lut-7-O-rutin on arthritis scores, body weight, spleen and thymus indices, and joint histopathology.

Our study not only identifies lut-7-O-rutin as a promising candidate molecule that warrants further investigation for the pharmacological treatment of RA but also offers mechanistic evidence supporting its therapeutic action.

## 2 Materials and methods

### 2.1 Isolation, culture, and identification of rat FLSs

Seven-week-old, female Sprague-Dawley (SD) rats were obtained from Shengchang (Shanghai, China). Animal experiments were approved by the Institutional Animal Care and Use Committee of Changzhou Second People’s Hospital (IACUC-2606001). Rats were anesthetized by intraperitoneal injection of 2.5% tribromoethanol at a dosage of 25 mL/kg, and synovial tissue was isolated from their knee joints using a dissecting microscope. The minced synovial tissue was then digested with 0.2% type II collagenase. Digested FLSs were cultured in high-glucose DMEM cell culture medium (Gibco, Gaithersburg, MD, United States) in an incubator containing 5% CO_2_ at 37 °C.

Rat FLSs were identified by immunofluorescence staining, which was carried out according to the manufacturer’s instructions (ABclonal, Wuhan, China). Briefly, cells were divided into anti-vimentin and anti-CD68 groups. Cells were seeded in 6-well plates and cultured until they reached 60%–80% confluence. The next day, cells were washed three times with PBS and fixed with 4% paraformaldehyde for 20 min. After washing three times with PBS, cells were permeabilized with a permeabilizing agent and blocked with 5% BSA at room temperature for 1 h. Samples were then incubated with primary antibodies against vimentin or CD68 overnight at 4 °C in a humidified chamber. The next day, samples were washed three times with PBS on a shaker, then incubated with the corresponding fluorescently labeled secondary antibodies at room temperature for 1 h in the dark and washed a further three times with PBS on a shaker. Finally, samples were mounted, sealed using an antifade mounting medium, and visualized under a fluorescence microscope (LSM 900, Carl Zeiss, Germany).

### 2.2 Drug toxicity assay of lut-7-O-rutin

A drug toxicity assay was carried out on the following three treatment groups: blank, untreated, and lut-7-O-rutin intervention. The blank group did not contain any cells, while FLSs were cultured overnight at a density of 5 × 10^3^ cells/well in a 96-well plate for the untreated and lut-7-O-rutin intervention groups. The next day, the medium in the blank and untreated groups was replaced with fresh culture medium, while the medium in the lut-7-O-rutin intervention group was replaced with culture medium containing different concentrations of lut-7-O-rutin: 0, 1.875, 3.75, 7.5, 15, and 30 µg/mL (MCE, Shanghai, China) with five replicate wells per concentration. Cells were incubated for 24 h in a cell incubator, after which 10 µL CCK-8 solution (Beyotime Biotechnology, Shanghai, China) was added to each well in the dark. Samples were incubated in the dark for 2 h, and the absorbance was then measured at 450 nm using a microplate reader (BioTek Epoch, USA). Based on these results, an optimal concentration of lut-7-O-rutin was selected for subsequent intervention experiments.

### 2.3 Enzyme-linked immunosorbent assay (ELISA) for measuring IL-6, TNF-α, and COX-2 levels in the supernatant of FLSs

We plan to select the optimal intervention concentration of lut-7-O-rutin by plotting dose-response curves. The experiment is divided into seven groups: control (normal FLSs); inflammation group (IL-1β-induced FLSs); lut-7-O-rutin treatment group (IL-1β-induced FLSs treated with 0.94 µg/mL lut-7-O-rutin); lut-7-O-rutin treatment group (IL-1β-induced FLSs treated with 1.88 µg/mL lut-7-O-rutin); lut-7-O-rutin treatment group (IL-1β-induced FLSs treated with 3.75 µg/mL lut-7-O-rutin); lut-7-O-rutin treatment group (IL-1β-induced FLSs treated with 7.50 µg/mL lut-7-O-rutin); and indomethacin (Indo) positive control group (IL-1β-induced FLSs treated with 4 µg/mL Indo). Cells (2 × 10^5^ cells/well) were cultured overnight in 6-well plates. The control and inflammation groups did not receive drug treatment, while the lut-7-O-rutin treatment and Indo positive control groups were treated with lut-7-O-rutin and Indo, respectively, for 24 h. Subsequently, the supernatant was collected from the treated cells. IL-6, TNF-α, and COX-2 concentrations in the supernatant of each treatment group were measured using the corresponding ELISA kits in accordance with the manufacturer’s instructions (Multisciences, Zhejiang, China). We selected the optimal intervention concentration of lut-7-O-rutin based on the dose-response curve, and then divided the samples into the following four groups to test its anti-inflammatory effect using the above-mentioned methods: control (normal FLSs); inflammation group (IL-1β-induced FLSs); lut-7-O-rutin treatment group (IL-1β-induced FLSs treated with 3.75 µg/mL lut-7-O-rutin); and Indo positive control group (IL-1β-induced FLSs treated with 4 µg/mL Indo).

### 2.4 Transcriptome sequencing and pathway enrichment analysis

Samples were divided into inflammation (IL-1β-induced FLSs) and lut-7-O-rutin treatment (IL-1β-induced FLSs treated with 3.75 µg/mL lut-7-O-rutin) groups. Each group comprised FLSs isolated from five different rats. After exposure to lut-7-O-rutin for 24 h, high-throughput transcriptome sequencing was performed on these two groups to screen for differentially expressed genes (adjusted P value <0.05, fold change >2), which were then subjected to Gene Ontology (GO) enrichment and Kyoto Encyclopedia of Genes and Genomes (KEGG) pathway analyses.

### 2.5 Western blot analysis

Total cellular protein was extracted from FLSs using RIPA buffer (Servicebio, Wuhan, China) containing protease and phosphatase inhibitors (Beyotime, Shanghai, China). Proteins were separated by SDS-PAGE and transferred onto nitrocellulose membranes. After blocking with 5% BSA at room temperature for 1 h, membranes were incubated overnight at 4 °C with the appropriate concentrations of the following primary rabbit antibodies: PI3K, p-PI3K, AKT, p-AKT, P65, p-P65, IκBα, p-IκBα, cyclin-dependent kinase 4 (CDK4), and cyclin D (CCND) (ABclonal, Wuhan, China). The next day, membranes were washed three times with Tris-buffered saline containing Tween 20 (TBST), then incubated with horseradish peroxidase (HRP)-conjugated goat anti-rabbit secondary antibodies at room temperature for 1 h. Finally, membranes were washed three times with TBST, and protein bands were visualized using ECL chemiluminescence (NCM Biotechnology, Suzhou, China). Glyceraldehyde 3-phosphate dehydrogenase (GAPDH) was used as the internal control.

### 2.6 Establishment of a rat CIA model to evaluate the therapeutic effects of lut-7-O-rutin *in vivo*

A CIA rat model was established using 7-week-old SD female rats (Shengchang, Shanghai, China) by first subcutaneously injecting a mixture of bovine type II collagen and complete Freund’s adjuvant (Chondrex Inc., Redmond, WA, USA) into the base of the tail. One week later, rats received a second immunization containing the same volume of bovine type II collagen and incomplete Freund’s adjuvant. Approximately 1 week after the second immunization, the arthritis score was determined in the CIA rats as follows: 0, no erythema or swelling; 1, erythema and mild swelling limited to the midfoot or ankle joint; 2, erythema and moderate swelling from ankle to foot; 3, erythema and swelling from the ankle to the metatarsal joints; and 4, erythema and severe swelling including ankle, foot, and toe. Two independent investigators interpreted the arthritis score, and if the scores were inconsistent, the two individuals discussed and resolved the issue. The threshold for successfully inducing CIA, as defined by the CIA scoring system, was a total score of ≥4 points across the four paws of the evaluated rats.

**FIGURE 1:**
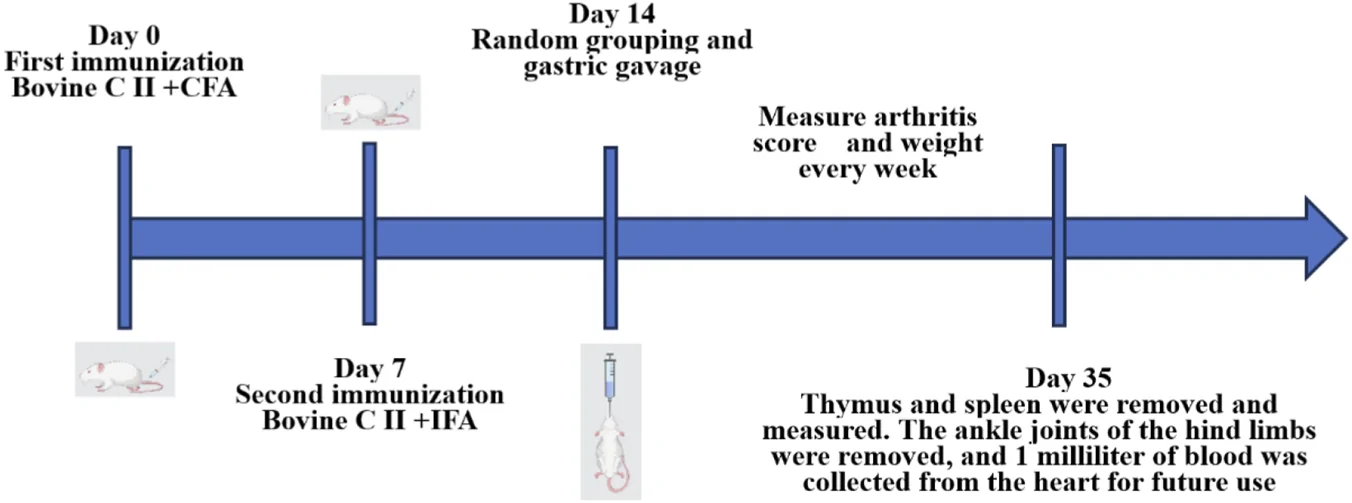
Schematic diagram outlining our *in vivo* intervention experiment. (C II: type II collagen; CFA: complete Freund’s adjuvant; IFA: incomplete Freund’s adjuvant. The mixed emulsion of C II and CFA was injected for the first immunization, and 1 week later, the mixed emulsion of C II and IFA was injected to strengthen the immunization. One week after successful CIA modeling, normal saline, luteolin-7-O-rutinoside (lut-7-O-rutin) and indomethacin (Indo) was administered by gavage once a day for 21 days).

The therapeutic effects of lut-7-O-rutin or Indo *in vivo* were then determined using the CIA rat model. Briefly, 12 female rats were randomly divided into the following four groups (3 rats per group): control (normal rats), CIA model, lut-7-O-rutin treatment (gastric gavage, 1 µg/g), and Indo positive control (gastric gavage, 1 µg/g). Lut-7-O-rutin and Indo were given by gastric gavage for 21 days, while the control and CIA model groups were given the same volume of physiological saline by gastric gavage for 21 days. The arthritis scores and body weights of the rats were recorded weekly from day 0 of gastric gavage. Twenty-four hours after the last gastric gavage, rats were weighed, then anesthetized by intraperitoneal injection of 2.5% tribromoethanol at a dosage of 25 mL/kg. The thymus and spleen were quickly removed from the anesthetized rats, rinsed with physiological saline, and the surface was gently dried with gauze. After weighing to obtain their wet weights, the organ index was calculated using the following formula: organ index = organ wet weight (mg)/experimental rat body weight (g) (Figure 1).

### 2.7 ELISA for measuring IL-6, TNF-a, and COX-2 levels in rat serum

Rats were anesthetized by intraperitoneal injection of 2.5% tribromoethanol at a dosage of 25 mL/kg 24 h after administration of the last gastric gavage, and 1 mL of blood was collected from the heart. The blood was left at room temperature for 30 min, then centrifuged at 1,000 × g for 10 min at 4 °C. Serum was extracted from the blood and stored in liquid nitrogen until further analysis. Commercially available ELISA kits (Multisciences, Zhejiang, China) were used to determine the concentrations of IL-6, TNF-α, and COX-2 in the rat serum.

### 2.8 Histopathological staining of the rat ankle joint

The ankle joints of the hind limbs of rats were fixed in 10% neutral buffered formaldehyde and sliced after decalcification, dehydration, and paraffin embedding. Tissue sections were stained with either hematoxylin and eosin (H&E; Servicebio, Wuhan, China) or Safranin O-Fast Green (Servicebio, Wuhan, China) according to the manufacturer’s instructions. Pathological changes in the ankle tissue were visualized using an optical microscope (Nikon, Japan). After the start of animal experiments, researchers who were not involved in subsequent histological evaluations were responsible. They randomized the experimental animals into groups, encoded them, and performed subsequent experimental procedures. In the histological grading stage, the histological investigator received slides with only a blind code for image collection and grading. The evaluation of the same section was performed by two independent investigators. If two investigators had inconsistent ratings, they resolved the issue through discussion. Pathological changes in the articular cartilage and synovium were evaluated and analyzed based on previously described scoring criteria (Wang et al., 2024). The specific histopathological scoring criteria of our study are as follows: (1) Synovial inflammation: 0 = normal, 1 = mild inflammatory cell infiltration, 2 = moderate infiltration, 3 = severe infiltration; (2) Cartilage erosion: 0 = normal, 1 = superficial erosion, 2 = moderate erosion reaching the middle layer of cartilage, 3 = full-thickness erosion. The scoring was performed blindly by two independent researchers, and consensus was reached through discussion in case of disagreements.

### 2.9 Immunohistochemistry (IHC)

IHC was performed according to the following procedure. Paraffin-embedded sections were first dewaxed using an environmentally friendly dewaxing solution in three graded baths (I–III), 10 min per bath, followed by sequential immersion in absolute ethanol (I–III), 5 min per bath, and then rinsed with distilled water. For antigen retrieval, sections were immersed in Tris-EDTA buffer (pH 9.0) and subjected to microwave heating (1000 W, 8 min), combined with pepsin treatment. After retrieval, sections were washed three times with PBS, 5 min each. Endogenous peroxidase activity was blocked by incubation with 3% H_2_O_2_ in the dark for 25 min, followed by blocking with 3% BSA at room temperature for 30 min. Primary antibodies against CDK4, p-AKT, and p-PI3K (ABclonal) were diluted 1:100 in PBS. After gently removing the blocking solution, the diluted primary antibodies were applied, and the sections were placed flat in a humidified chamber and incubated overnight at 4 °C. Subsequently, the sections were incubated with an S-vision immunohistochemical polymer secondary antibody (goat anti-rabbit). DAB staining was monitored under a microscope, and brownish-yellow staining was considered a positive signal. Nuclei were counterstained with hematoxylin for 3 min, followed by differentiation and bluing. Sections were then dehydrated through graded ethanol solutions (75% → 85% → 100% → 100%), n-butanol, and xylene, each for 5 min, and mounted after air drying. Sections were examined under a light microscope (Nikon Instruments Inc., E100). The results were evaluated by two independent observers. When the two observers agreed, the result was recorded; in cases of disagreement, a consensus was reached after discussion. For each sample, five randomly selected high-power fields (400×) were analyzed, and positive cells were counted using ImageJ software. Data visualization was performed using GraphPad Prism 8.0.2. (GraphPad Software, United States).

### 2.10 Statistical analysis

All data were first subjected to the Shapiro-Wilk normality test and Levene’s test for homogeneity of variances. Data satisfying normal distribution and homogeneity of variances were analyzed using one-way analysis of variance (one-way ANOVA), and Tukey’s multiple comparison test was adopted for pairwise intergroup comparisons. Repeated measurement data (such as body weight and arthritis scores) were analyzed via two-way analysis of variance (two-way ANOVA), with Bonferroni’s *post hoc* test applied for intergroup comparisons. All data are presented as the mean ± standard deviation using GraphPad Prism 8.0.2. A *P* value <0.05 was considered statistically significant.

## 3 Results

### 3.1 Acquisition and identification of rat FLSs

**FIGURE 2:**
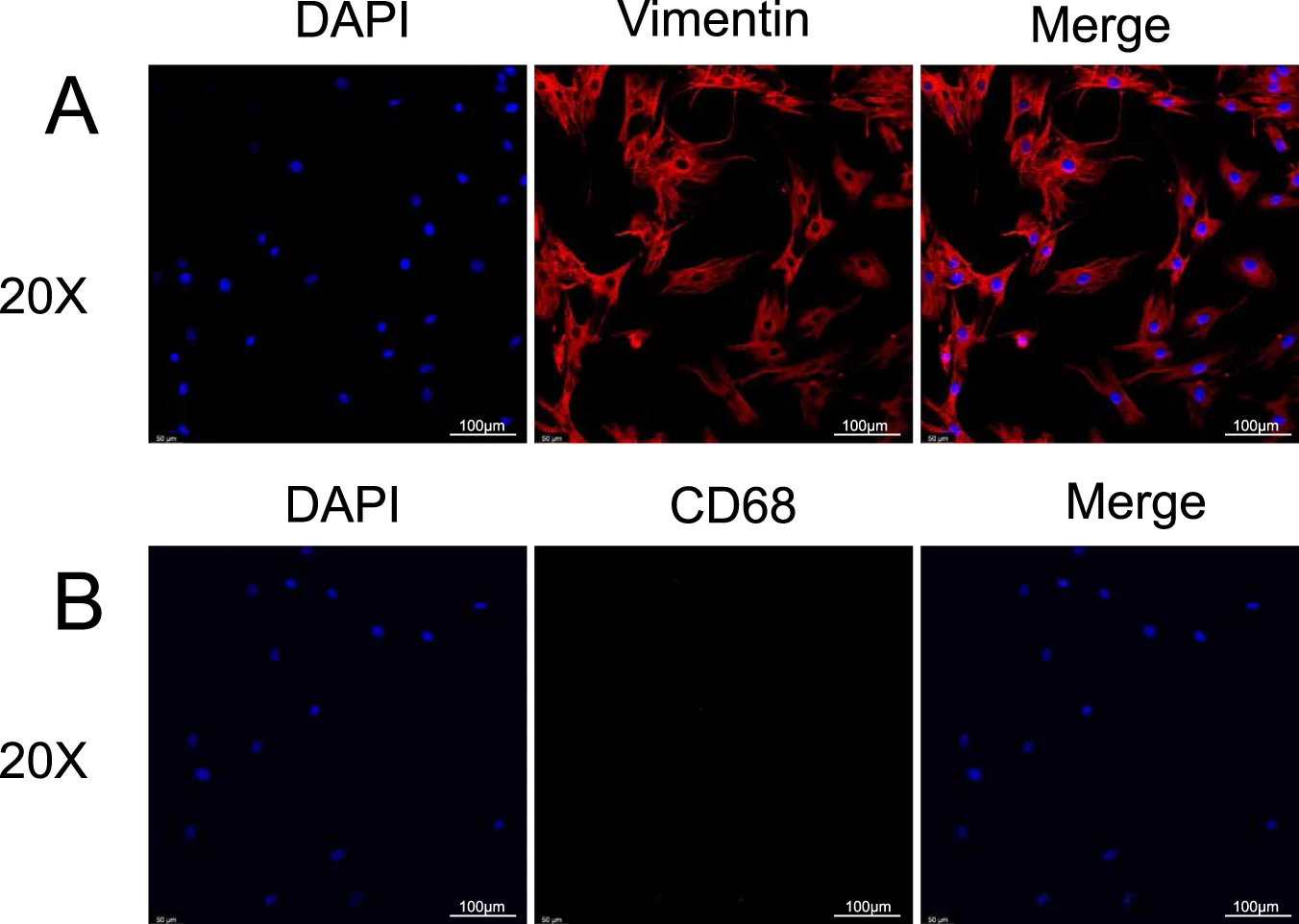
Identification of FLSs in the knee joint of normal rats by immunofluorescence staining. **(A)** Red fluorescence indicates positive staining for vimentin; **(B)** Green fluorescence indicates negative staining for CD68.

FLSs were successfully isolated from rat knee joints, as verified by immunofluorescence staining, which confirmed that the isolated cells stained positive for vimentin and negative for CD68 (Figure 2).

### 3.2 Drug toxicity testing to determine the optimal concentration of lut-7-O-rutin for subsequent experiments

**FIGURE 3:**
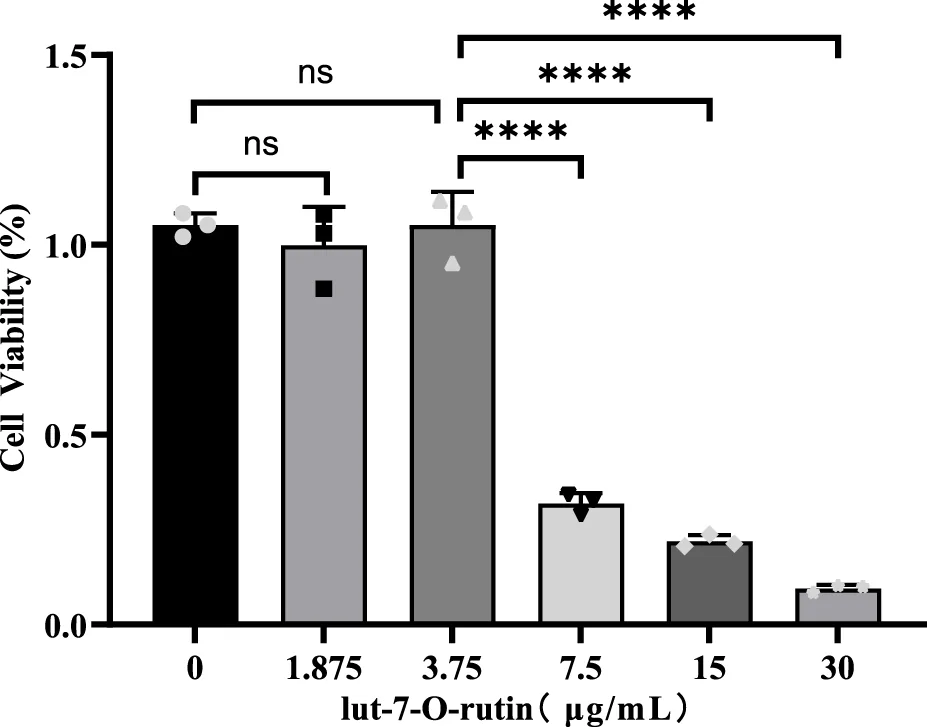
Cell viability of normal rat FLSs treated with different concentrations (0, 1.875, 3.75, 7.5, 15, 30 μg/mL) of lut-7-O-rutin for 24 h. One-way ANOVA. ****P < 0.0001; ns: no significance. Data are presented as mean ± standard deviation (mean ± SD), n = 3.

Our drug toxicity test data demonstrate that when the concentration of lut-7-O-rutin is increased to 7.5 μg/mL, the viability of rat FLSs decreases markedly, with the cell survival rate dropping significantly to 31.3% (*P* < 0.0001). At a concentration of 3.75 μg/mL, the cell survival rate reaches 99.4% of that in the control group, showing no statistically significant difference compared with the control group (*P* > 0.05). Accordingly, lut-7-O-rutin at 3.75 μg/mL was selected for subsequent intervention experiments in this study (Figure 3).

### 3.3 Lut-7-O-rutin inhibits IL-1β-induced inflammation in FLSs

**FIGURE 4:**
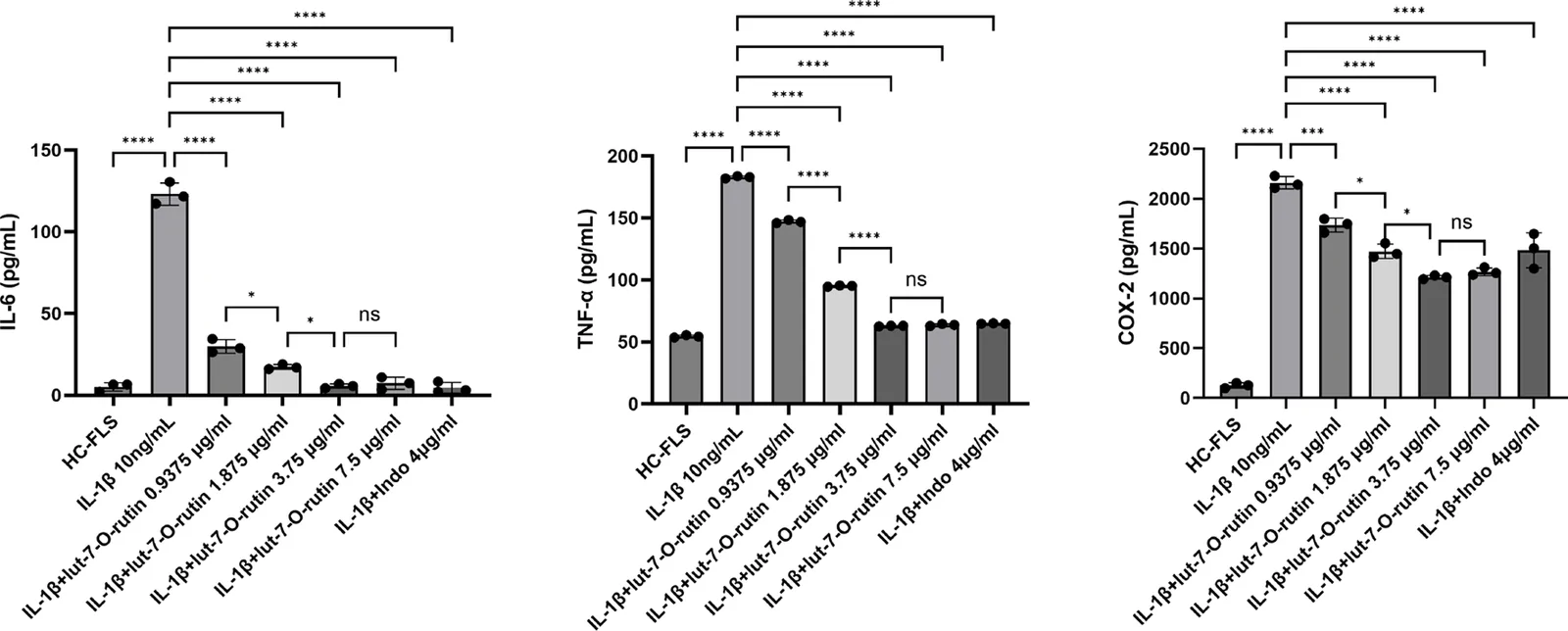
The dose-response curve of lut-7-O-rutin intervention treatment was detected using the ELISA method. The figure shows that after lut-7-O-rutin intervention, the production of IL-6, TNF-α and COX-2 in cell supernatants exhibited a dose-dependent manner, with a concentration of 3.75 μg/mL serving as the optimal intervention concentration. One-way ANOVA. *P < 0.05, ***P < 0.001, ****P < 0.0001. Data are presented as mean ± SD, n = 3.

**FIGURE 5:**
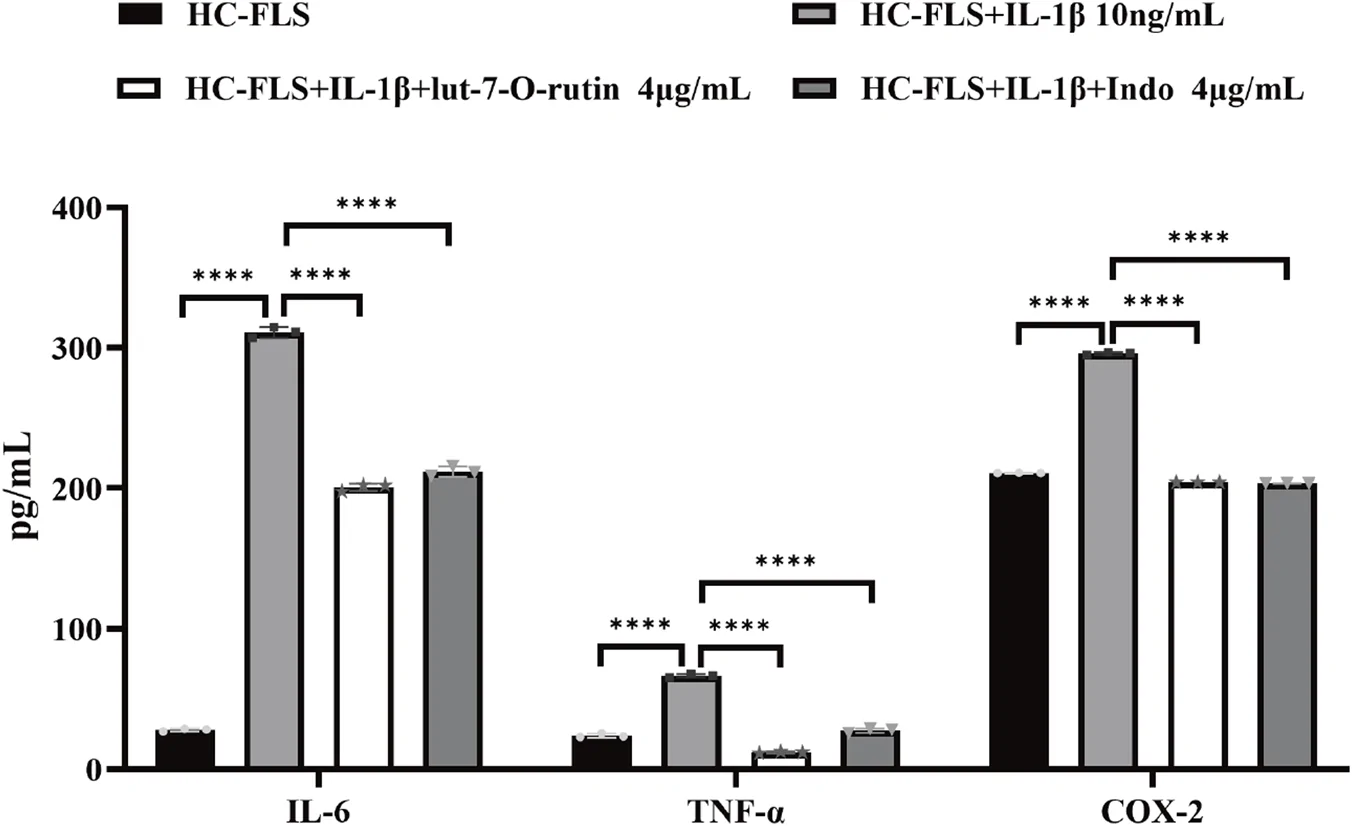
The effects of lut-7-O-rutin on the production of IL-6, TNF-α and COX-2 in cell supernatants were detected by ELISA. One-way ANOVA. ****P < 0.0001. Data are presented as mean ± SD, n = 3.

The results detected by ELISA showed that the intervention effect of lut-7-O-rutin was concentration-dependent, with 3.75 μg/mL as the optimal intervention concentration (Figure 4). We further conducted intervention experiments using lut-7-O-rutin at a concentration of 3.75 μg/mL, and the results indicated that the levels of inflammatory mediators (TNF-α, IL-6, and COX-2) in the inflammation group were significantly higher than in the control (HC-FLS) group as measured by ELISA (Figure 5). However, a significant reduction in the concentrations of inflammatory mediators was observed in the lut-7-O-rutin treatment and Indo positive control groups compared with the inflammation group (Figure 5). These findings suggest that lut-7-O-rutin (3.75 μg/mL) significantly inhibits the IL-1β-induced inflammatory response in FLSs.

### 3.4 Transcriptome sequencing and pathway enrichment analyses

**FIGURE 6:**
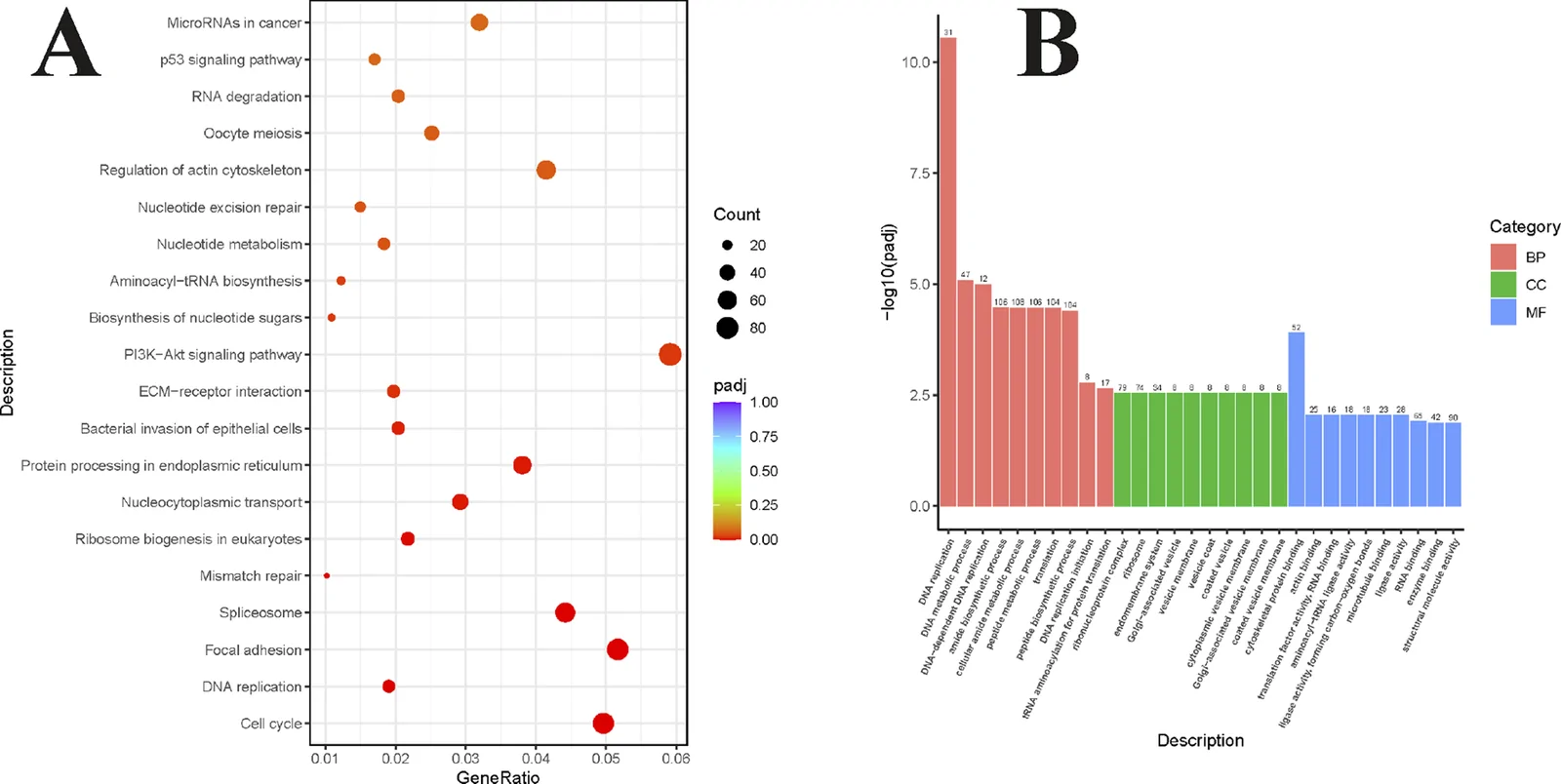
GO enrichment and KEGG pathway analysis. **(A)** GO enrichment analysis showing the biological processes (BPs) significantly associated with the therapeutic effects of lut-7-O-rutin. **(B)** KEGG pathway enrichment analysis identifying the significantly downregulated pathways associated with the therapeutic effects of lut-7-O-rutin.

Transcriptome sequencing analysis identified 373 differentially expressed genes, including 269 downregulated genes and 104 upregulated genes. GO enrichment analysis revealed a significant correlation between differential gene expression and the following biological processes (BP): DNA replication, DNA metabolic processes, and DNA-dependent DNA replication; cellular components (CC): ribonucleoprotein complex, ribosome, endomembrane system, and cytoskeletal protein binding; and molecular functions (MF): actin binding, translation factor activity, and RNA binding (Figure 6A). KEGG pathway enrichment analysis showed that pathways such as the PI3K/AKT and cell cycle signaling pathways were significantly downregulated (Figure 6B).

### 3.5 Effects of lut-7-O-rutin on the PI3K/AKT/NF-kB and cell cycle signaling pathways

**FIGURE 7:**
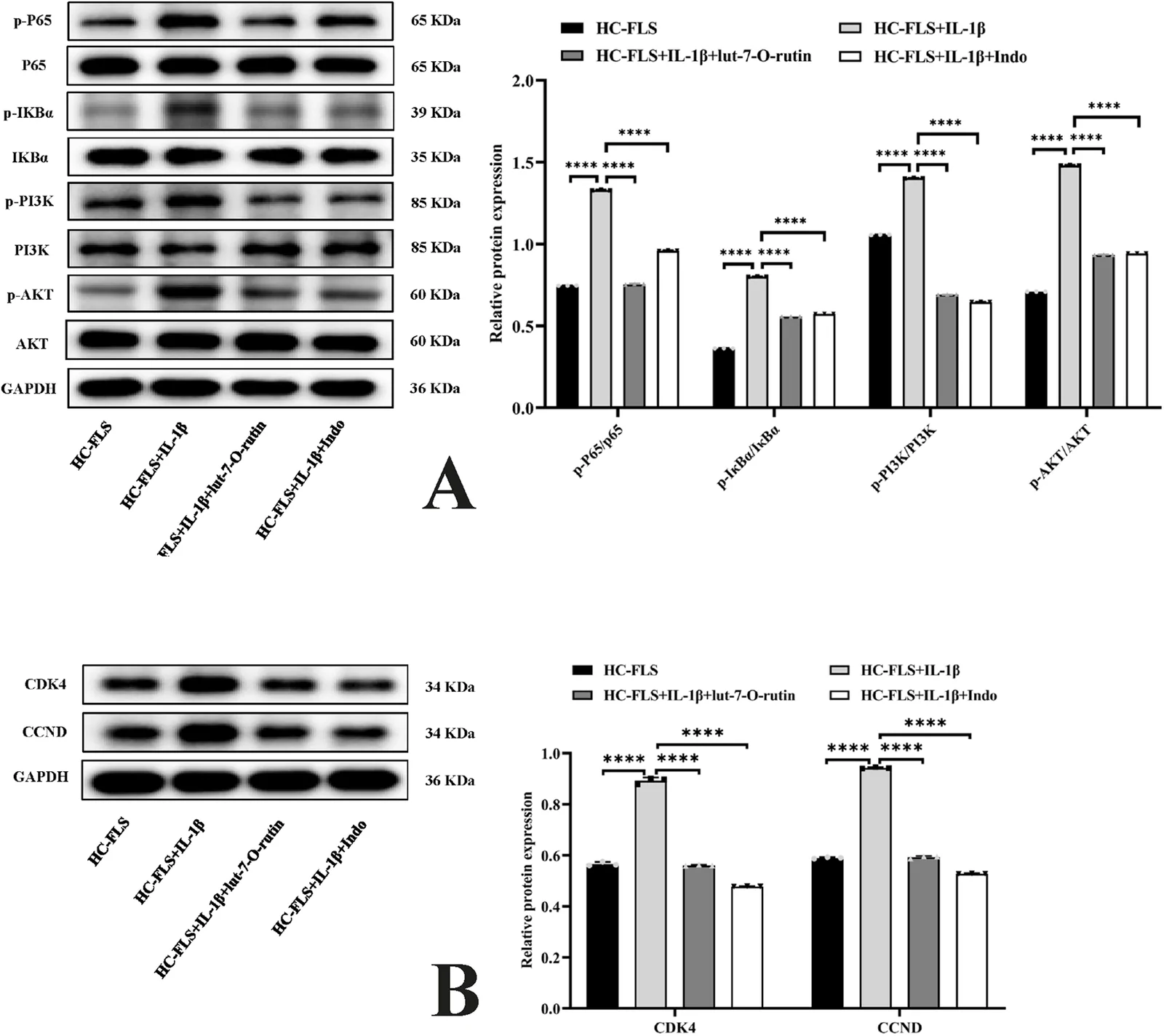
Western blot experiment detects the expression of key proteins in the pathways. **(A)** Lut-7-O-rutin significantly inhibited the phosphorylation levels of the pathway of CIA-FLS. The effect of lut-7-O-rutin on NF-κB/PI3K/AKT signaling pathway was detected by Western blot. Lut-7-O-rutin significantly inhibited the phosphorylation levels of p-P65, p-IκBα, p- PI3K and p-Akt. One-way ANOVA. Data are presented as mean ± SD, n = 3. **(B)** Lut-7-O-rutin significantly inhibited the abnormal proliferation of FLSs induced by IL-1b. Western blot was used to detect the effects of lut-7-O-rutin on the expression levels of cyclin-dependent kinase 4 (CDK4) and cyclin D (CCND), key factors in cell cycle signaling pathways. One-way ANOVA. ****P < 0.0001. Data are presented as mean ± SD, n = 3.

We found that the phosphorylation levels of key components of the PI3K/AKT/NF-κB pathway were significantly higher in the inflammation group than in the control (HC-FLS) group. Similarly, the expression levels of CDK4 and CCND, key components of the cell cycle signaling pathway, were significantly higher in the inflammation group than in the control (HC-FLS) group. Treatment of IL-1β-induced FLSs with lut-7-O-rutin or the Indo positive control led to a significant reduction in the phosphorylation levels of key components of the PI3K/AKT/NF-κB pathway compared with the inflammation group (Figure 7A), as well as a significant reduction in CDK4 and CCND expression levels (Figure 7B). Our findings indicate that lut-7-O-rutin may exert anti-inflammatory therapeutic effects by inhibiting the PI3K/AKT/NF-κB pathway, as well as inhibit the abnormal proliferation of IL-1β-induced FLSs by suppressing the cell cycle signaling pathway.

### 3.6 Gastric gavage of lut-7-O-rutin significantly eases arthritis in CIA rats

**FIGURE 8:**
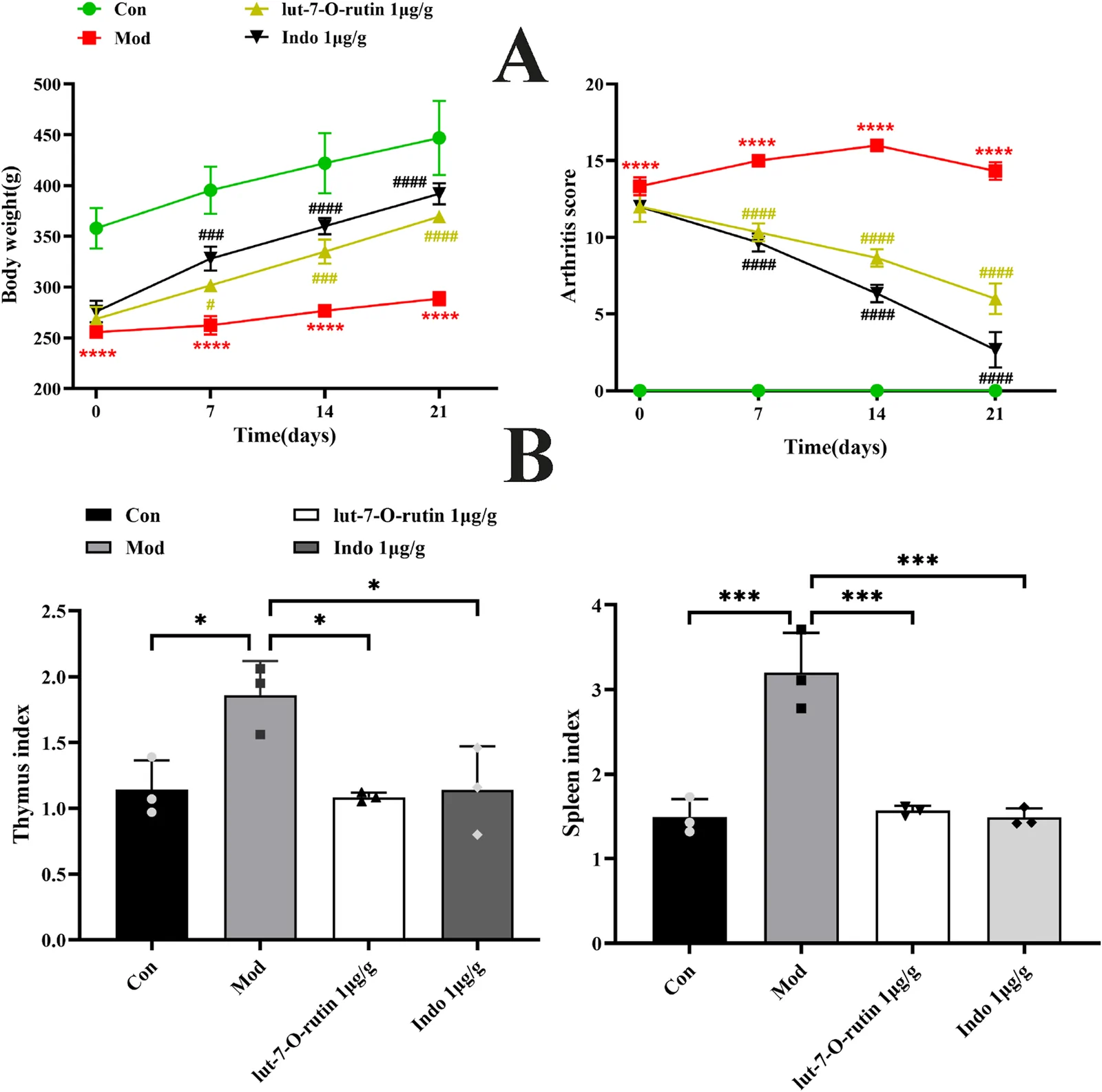
Changes in body weight, arthritis score, and organ index after Lut-7-O-rutin intervention in CIA rats. **(A)** Lut-7-O-rutin inhibits the development of arthritis in CIA rats. Effects of lut-7-O-rutin on body weight and arthritis score of CIA rats at different time points. After treatment with lut-7-O-rutin and Indo, the body weight of rats increased significantly on the seventh, 14th and 21st days after gavage. The arthritis score of rats began to decrease significantly after 1 week of gavage. Two-way ANOVA. *: compared with the control group (Con); ^#^: compared with the model group (Mod). ^****^ *P* < 0.0001, ^#^ *P* < 0.05, ^###^ *P* < 0.001, ^####^ *P* < 0.0001. Data are presented as mean ± SD, n = 3. **(B)** Weight changes of thymus and spleen in CIA rats after gastric gavage. After treatment with lut-7-O-rutin and Indo, the organ index of rats decreased significantly. One-way ANOVA. ^*^ *P* < 0.05, ^***^ *P* < 0.001. Data are presented as mean ± SD, n = 3.

We successfully established a model of CIA in rats, as indicated by redness and swelling of the hind limbs of the CIA rats, together with an arthritis score greater than 4. The body weights of normal control rats gradually increased throughout the experiment. The body weights of lut-7-O-rutin-treated rats were significantly higher than those of CIA rats 1 week after gastric gavage administration of lut-7-O-rutin (*P <* 0.05). Indeed, rats in the lut-7-O-rutin treatment and Indo positive control groups showed significant weight gain on the seventh, 14th, and 21st days after gastric gavage compared with CIA rats (Figure 8A). At the same time, the arthritis scores of rats in the lut-7-O-rutin treatment and Indo positive control groups were significantly lower than those of CIA rats from 1 week after gastric gavage (*P <* 0.0001), suggesting that lut-7-O-rutin significantly alleviates the symptoms of arthritis in CIA rats (Figure 8A). Finally, the organ indices (thymus and spleen) of CIA rats were found to be significantly higher than those of normal control rats (*P <* 0.05), while the organ indices (thymus and spleen) of rats in the lut-7-O-rutin treatment and Indo positive control groups were found to be significantly lower than those in CIA rats (*P <* 0.001) (Figure 8B).

### 3.7 Effects of lut-7-O-rutin on inflammatory mediator levels in the serum of CIA rats

**FIGURE 9:**
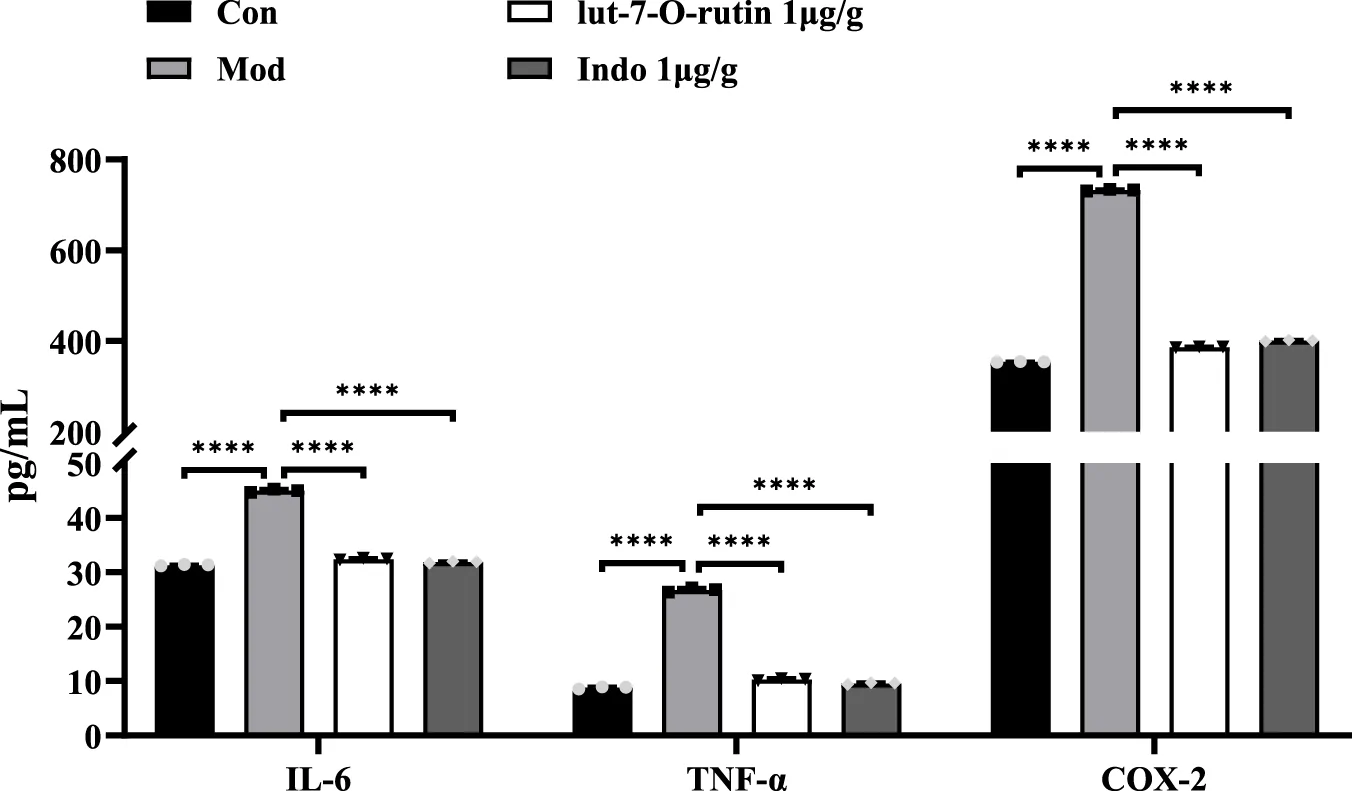
Concentrations of IL-6, TNF-a and COX-2 in the serum of rats in the normal control (Con), CIA model (Mod), lut-7-O-rutin treatment, and Indo positive control groups assessed by ELISA. One-way ANOVA. ^****^ *P* < 0.0001. Data are presented as mean ± SD, n = 3.

IL-6, TNF-α, and COX-2 concentrations were found to be significantly increased in the serum of CIA rats compared with normal control rats (*P <* 0.0001), while IL-6, TNF-α, and COX-2 concentrations in the serum of lut-7-O-rutin treatment and Indo positive control rats were found to be significantly lower than those in CIA rats (*P <* 0.0001) (Figure 9).

### 3.8 Histological analysis of the effects of lut-7-O-rutin on the ankle joints of CIA rats

**FIGURE 10:**
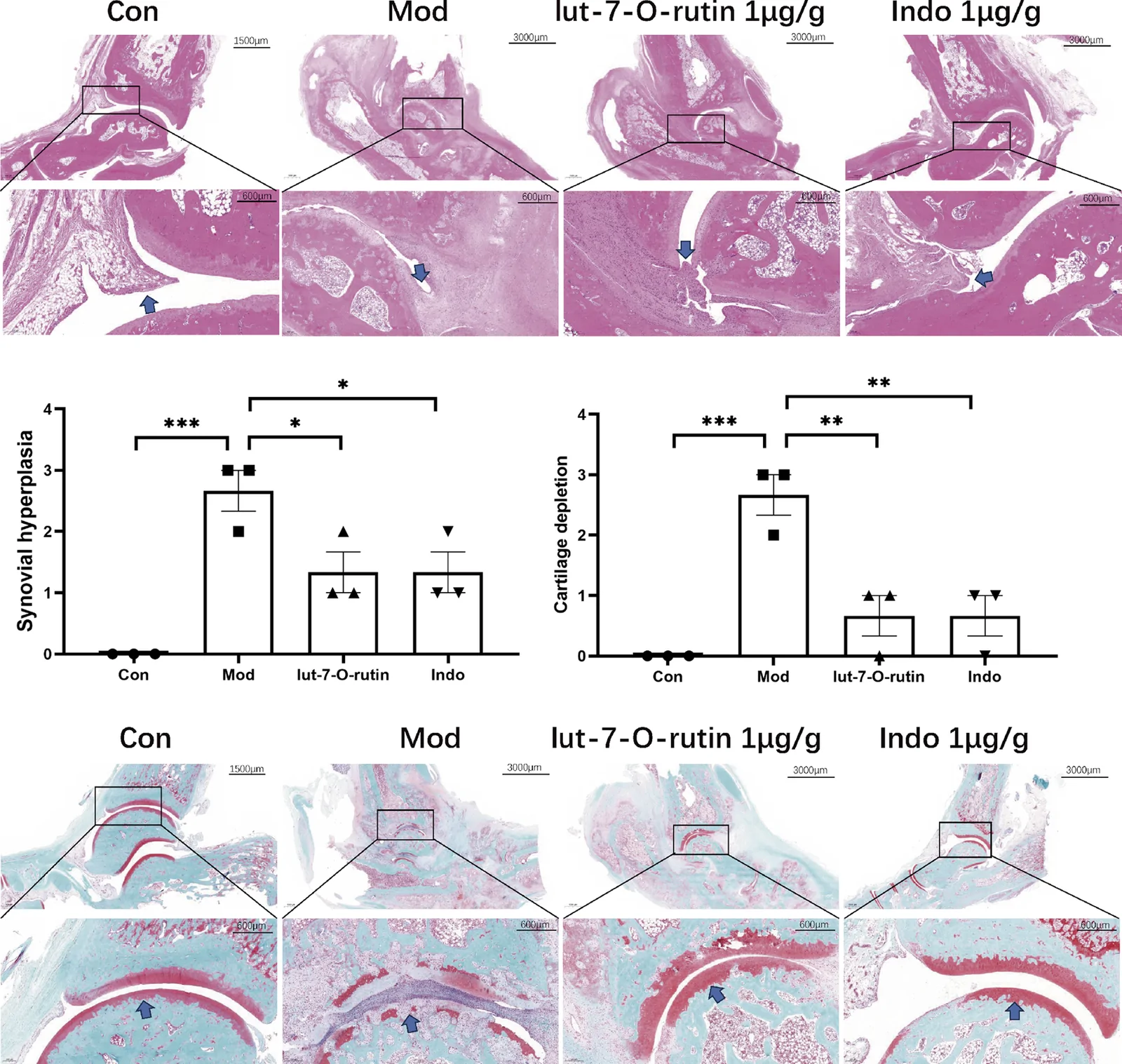
H&E and Safranin O-Fast Green staining of rat ankle tissue. After treatment with lut-7-O-rutin and Indo, the thickness of synovial epithelium decreased and the infiltration of inflammatory cells was inhibited, which also improved cartilage damage. One-way ANOVA. ^*^ *P* < 0.05, ^**^ *P* < 0.01, ^***^ *P* < 0.001. Data are presented as mean ± SD, n = 3.

Our histopathological data showed that the synovial membrane of the ankle joint in normal control rats was thin and smooth, with uniform thickness. In contrast, the synovial membrane of the ankle joint in CIA rats was thickened with a large amount of inflammatory cell infiltration. Rats in the lut-7-O-rutin treatment and Indo positive control groups showed a significant reduction in synovial hyperplasia and inflammatory cell infiltration in the ankle joint compared with CIA rats (Figure 10).

The ankle joints of rats were stained with Safranin O-Fast Green to visualize cartilage (red) and bone tissue (green). The ankle joint cavity structure of the normal control rats was intact, the joint space was uniform, and the cartilage surface was smooth. Rats in the CIA model group showed synovial hyperplasia of the ankle joint, cartilage destruction, narrowing of the joint cavity, and a rough cartilage surface compared with normal control rats. A significant reduction in inflammation, cell infiltration, and cartilage damage was observed in the ankle joints of lut-7-O-rutin treatment and the Indo positive control rats compared with CIA model rats (Figure 10).

### 3.9 IHC analysis of the effects of lut-7-O-rutin on the ankle joints of CIA rats

**FIGURE 11:**
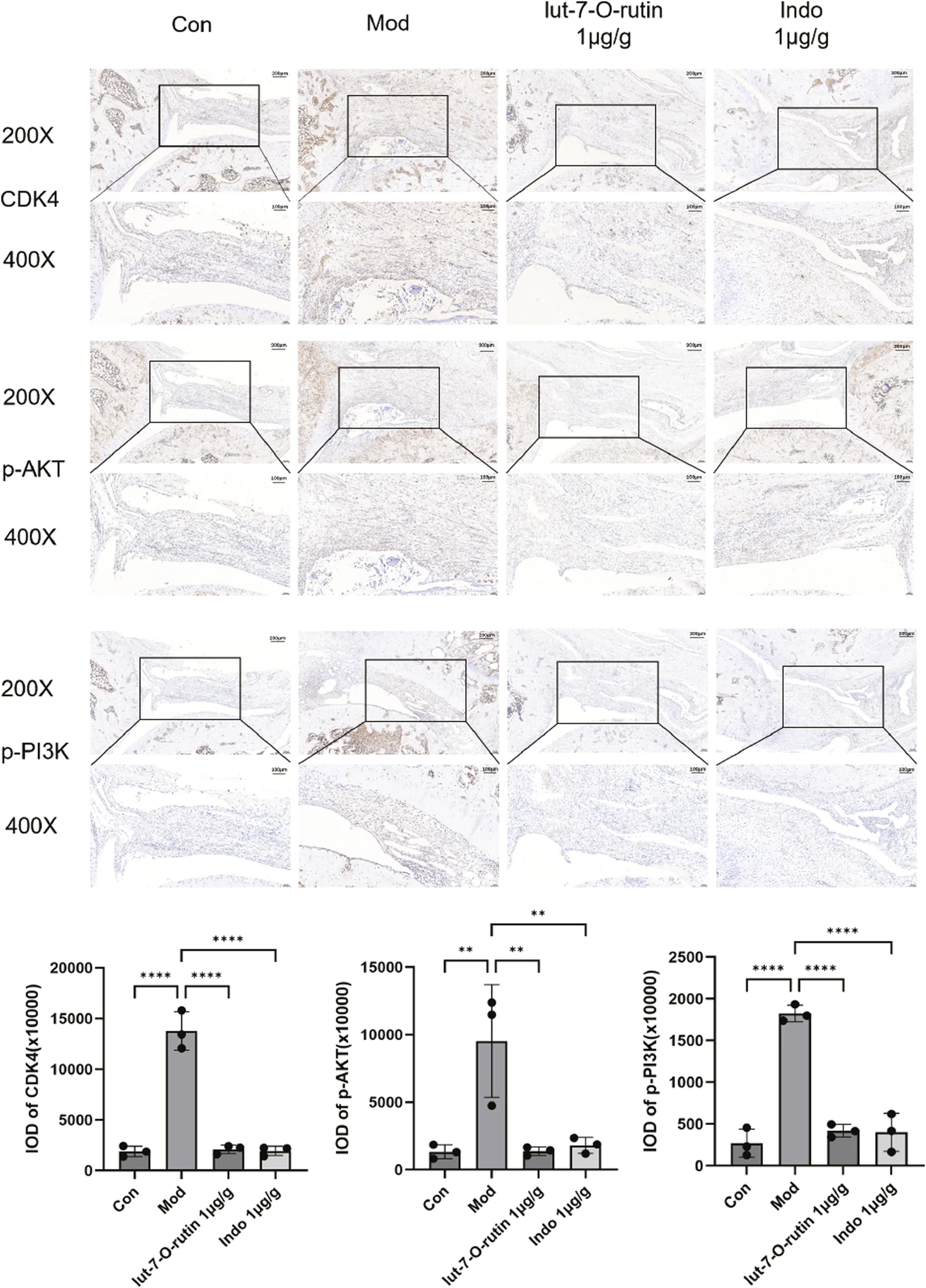
Immunohistochemical staining revealed that lut-7-O-rutin and Indo treatment significantly decreased the expression of p-PI3K, p-AKT and CDK4 in the ankle joints of CIA rats. One-way ANOVA. **P < 0.01, ****P < 0.0001. Data are presented as mean ± SD, n = 3.

The protein expression levels of p-PI3K, p-AKT, and CDK4 in the ankle joints of rats were evaluated by immunohistochemical analysis (Figure 11). Compared with the Con group, the expression levels of p-PI3K, p-AKT, and CDK4 were significantly increased in the Mod group (*P <* 0.0001, *P <* 0.01, and *P <* 0.0001). Following treatment with lut-7-O-rutin, the expression levels of p-PI3K, p-AKT, and CDK4 were significantly decreased in the lut-7-O-rutin group (*P <* 0.0001, *P <* 0.01, and *P <* 0.0001). Similarly, the Indo positive control group also exhibited significantly reduced expression of p-PI3K, p-AKT, and CDK4 (*P <* 0.0001, *P <* 0.01, and *P <* 0.0001). Taken together, these data indicate that lut-7-O-rutin alleviates arthritis in CIA rats by downregulating key proteins involved in the PI3K/AKT and cell cycle signaling pathways.

## 4 Discussion

RA is a chronic autoimmune inflammatory disease. The abnormal proliferation and invasion of FLSs are important contributors to the pathogenesis of RA (McInnes and Schett, 2011). Currently, studies examining the association between luteolin and RA have been predominantly focused on luteolin itself, with virtually no research dedicated to understanding the effects of its naturally occurring glycoside form (lut-7-O-rutin). Glycosylation of luteolin can alter its pharmacokinetic properties, such as absorption and distribution, leading to increased water solubility and bioavailability (Cunha et al., 2026; Morand et al., 2000; Slámová et al., 2018). In the current study, we treated IL-1β-induced FLSs with lut-7-O-rutin and found that lut-7-O-rutin possesses significant anti-inflammatory effects. Transcriptome sequencing revealed, and Western blotting confirmed, that these anti-inflammatory effects may be mediated through inhibition of the PI3K/AKT/NF-κB pathway and regulation of the cell cycle signaling pathway. Treatment of CIA rats with lut-7-O-rutin by gastric gavage led to a significant reduction in the arthritis score and organ indices of CIA rats. Together, these findings suggest that lut-7-O-rutin has therapeutic potential in CIA rats.

Lut-7-O-rutin is a natural flavonoid compound formed by the combination of luteolin and a disaccharide moiety (rutinose, composed of rhamnose and glucose). Glycosylation enhances its water solubility and bioavailability, as well as its biological activity. Lut-7-O-rutin is found in various medicinal and edible plants, such as honeysuckle, mint, wild chrysanthemum, and olive leaves, and is also one of their main active constituents (Omar, 2010; Hudz et al., 2023; Gao et al., 2024). Some studies have shown that lut-7-O-rutin can improve insulin resistance and regulate blood glucose metabolism in the treatment of diabetes (Subash-Babu et al., 2023; Fecka et al., 2023). Furthermore, in the treatment of inflammatory diseases, luteolin has been shown to inhibit the expression of various pro-inflammatory mediators, such as TNF-α, IL-1β, and IL-6, by suppressing the MAPK and NF-κB signaling pathways (Guo et al., 2025; Xia et al., 2014; Zhang et al., 2017). Recently, the ethanol extract of *Teucrium montanum* L., which possesses anti-inflammatory and immunomodulatory properties, was found to contain mainly verbascoside and lut-7-O-rutin (Bufan et al., 2024).

Here, we confirmed that treatment of IL-1β-induced FLSs with lut-7-O-rutin significantly reduced inflammatory mediators such as TNF-α, IL-6, and COX-2, highlighting its significant anti-inflammatory effects. We further examined its mechanism of action by performing transcriptome sequencing on the lut-7-O-rutin treatment and inflammation groups. We found that genes that were significantly downregulated after lut-7-O-rutin treatment were enriched in the PI3K/AKT and cell cycle signaling pathways. Our Western blotting data further showed that lut-7-O-rutin reduced the production of inflammatory mediators and inhibited the inflammatory response of IL-1β-induced FLSs by inhibiting the PI3K/AKT/NF-κB signaling pathway.

The synovial membrane of RA patients often exhibits abnormal proliferation, which has invasive and destructive effects (Lee et al., 2023; Lu et al., 2022). Here, we showed by Western blotting that lut-7-O-rutin inhibits CDK4 and CCND, key components of the cell cycle signaling pathway, thereby suppressing abnormal proliferation of FLSs and slowing down disease progression.

The CIA rat model can effectively simulate the inflammation, synovial hyperplasia, and bone destruction characteristic of RA. Here, we administered gastric gavage therapy to CIA rats and established both negative and positive controls. We found that gastric gavage with lut-7-O-rutin led to a reduction in the degree of joint redness and swelling in CIA rats, as well as a significantly lower arthritis score. Analysis of rat serum after gastric gavage revealed that the levels of inflammatory mediators in the lut-7-O-rutin treatment group were significantly lower than those in untreated CIA rats. The positive control group treated with Indo by gastric gavage showed similar therapeutic effects. Histopathological analysis of the ankle joint with H&E and Safranin O-Fast Green staining revealed less synovial inflammation, synovial hyperplasia, cartilage erosion, and subchondral bone destruction in the ankle joints of rats from the lut-7-O-rutin group than in the CIA model group. Our analysis of IHC results showed that lut-7-O-rutin significantly inhibited the expression of key proteins in the PI3K/AKT and cell cycle signaling pathways. These results suggest that lut-7-O-rutin has a significant therapeutic effect on joint inflammation in CIA rats.

There are relatively few studies focusing on lut-7-O-rutin, and limited literature data are available regarding its gastric gavage in rats. After reviewing relevant publications on intragastric luteolin delivery in rats, we found that the commonly adopted dosage regimens for intragastric luteolin administration range from 0.3 to 100 mg/kg (Abu-Elsaad and El-Karef, 2017; Abu-Elsaad and El-Karef, 2019; Madhesh and Vaiyapuri, 2013). Based on the concentrations used in our *in vitro* assays, the extrapolated *in vivo* dosage is 0.0375 μg/g, which can serve as an extremely low initial reference dose. Compared with luteolin, glycosylated lut-7-O-rutin exhibits improved water solubility and enhanced intestinal absorption. Taking all the above factors into consideration, our research group selected a daily intragastric dosage of 1 μg/g for rats. The outcomes of our intragastric administration experiment indicate that lut-7-O-rutin markedly alleviates inflammation in CIA rats.

Although lut-7-O-rutin has broad therapeutic potential, its development into a mature therapeutic drug still faces challenges. Future studies will focus on improving its bioavailability. Since flavonoid glycosides need to be hydrolyzed by microorganisms in the intestine before they can be absorbed, future research directions will include the development of nano-formulations, phospholipid complexes, or structural modifications to enhance their absorption and bioavailability. In addition, since the majority of studies are currently limited to cellular and animal models, there is a critical need to develop well-designed and large-scale clinical studies to validate its effectiveness, safety, and appropriate dosage in humans. Finally, further studies are required to determine the underlying mechanisms of its multi-target synergistic effects, specifically to further elucidate its mechanism of action in complex disease networks and its ability to coordinate multiple signaling pathways to achieve therapeutic effects.

There are several limitations associated with this study. (1) The pathogenesis of RA is complex and may involve multiple signaling pathways. However, in the current study, only two signaling pathways associated with RA were examined. (2) This experiment was conducted using rat-derived FLSs and CIA rat models, and the findings have not yet been validated in human FLSs or human RA synovial tissues. In the future, clinical RA specimens and patient-derived samples will be collected to further investigate the effects and mechanisms of lut-7-O-rutin in patients with RA. (3) Our experiment mainly focuses on the pharmacodynamic study of lut-7-O-rutin, while its pharmacokinetic research is still insufficient and needs to be further improved in the future.

Furthermore, the interpretation of the *in vitro* mechanistic data warrants particular caution due to the inherent properties of the flavonoid scaffold. Luteolin and its glycosides belong to a chemical class frequently flagged as pan-assay interference compounds (PAINS). These polyphenolic structures can act as potent aggregators, redox cyclers, or metal chelators, leading to non-specific inhibition of enzymes or receptors and generating false-positive readouts across a wide range of biochemical and cell-based assays (Baell and Holloway, 2010; Jasial et al., 2017). Therefore, it is plausible that the observed reductions in inflammatory mediators and phospho-proteins in IL-1β-induced FLSs are, to some extent, compounded by simple assay interference rather than purely specific on-target pathway modulation, and there is nothing wrong about stating this openly (Li et al., 2026; Bolz et al., 2021).

To mitigate this critical issue and establish a better evidence hierarchy, we employed a multi-method approach that intentionally prioritizes *in vivo* validation. According to this framework, the strongest tier of evidence derives from our CIA rat model: the significant attenuation of arthritis scores, histopathological joint protection (H&E and Safranin O-Fast Green), and the reduction of serum inflammatory cytokines (TNF-α, IL-6, COX-2) by lut-7-O-rutin gastric gavage are *in vivo* pharmacological effects unencumbered by typical *in vitro* PAINS artifacts. The second tier of substantiating evidence is the *in situ* IHC analysis of the rat ankle joints, which independently confirmed the downregulation of p-PI3K, p-AKT, and CDK4 in the target tissue, thereby linking the histological improvement directly to the proposed pathways without reliance on cultured cells. The third tier, the *in vitro* transcriptome profiling, provides a global, unbiased snapshot suggesting that the PI3K/AKT and cell cycle axes are the most prominently downregulated pathways; while still an *in vitro* endpoint, it is less susceptible to certain types of interference than single-concentration immunoassays. The weakest tier is the targeted cell-based ELISA and Western blot data, which, in isolation, could indeed represent a simple pan-assay interference. By leveraging this hierarchical structure, where the top-tier *in vivo* and *in situ* data independently recapitulate the cellular and transcriptomic findings, the likelihood that our core conclusion is merely an artifact is substantially reduced, allowing for a balanced and more realistic evaluation of the compound’s pharmacological potential.

**FIGURE 12:**
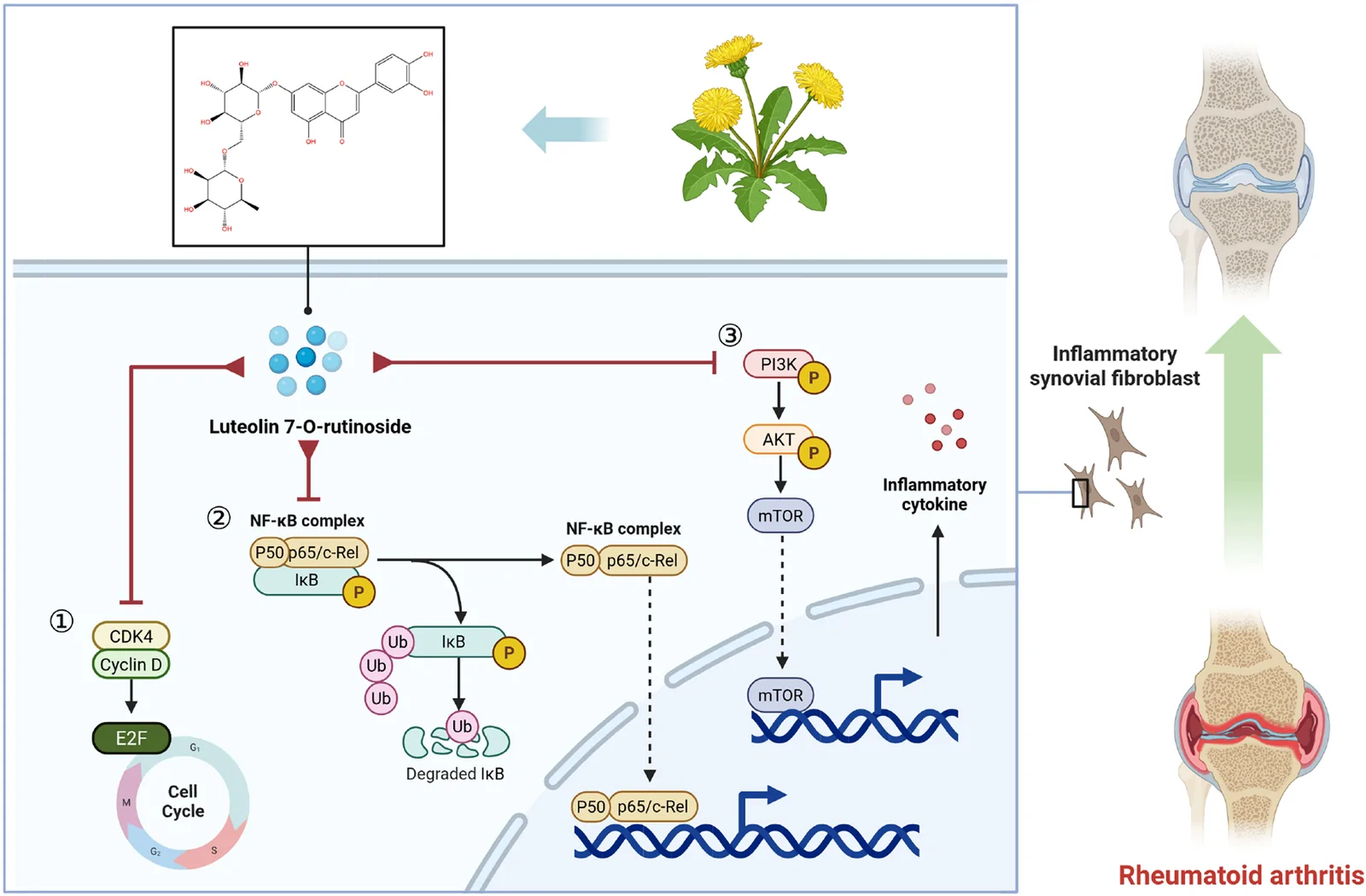
Lut-7-O-rutin alleviates the progression of RA by inhibiting the PI3K/AKT/NF-kB and cell cycle signaling pathways.

In conclusion, our *in vitro* and *in vivo* experiments have confirmed that lut-7-O-rutin can exert therapeutic effects on RA may be mediated through inhibiting the PI3K/AKT/NF-κB and cell cycle signaling pathways (Figure 12). Lut-7-O-rutin is a natural compound with multi-target and multi-pathway pharmacological activity, which shows great potential in anti-inflammatory and anti-arthritic therapy. However, to successfully translate our laboratory research findings into clinical applications, breakthroughs still need to be made in drug delivery systems and clinical validation.

## Data Availability

The data presented in the study are deposited in the Sequence Read Archive repository, accession number PRJNA1507873.
